# Pain Associated with Wound Care Treatment among Buruli Ulcer Patients from Ghana and Benin

**DOI:** 10.1371/journal.pone.0119926

**Published:** 2015-06-01

**Authors:** Marike Alferink, Janine de Zeeuw, Ghislain Sopoh, Chantal Agossadou, Karibu M. Abass, Richard O. Phillips, Susanne Loth, Emma Jutten, Yves T. Barogui, Roy E. Stewart, Tjip S. van der Werf, Ymkje Stienstra, Adelita V. Ranchor

**Affiliations:** 1 Department of Health Psychology, University Medical Center Groningen, University of Groningen, P.O. Box 196, 9700 AD, Groningen, The Netherlands; 2 Department of Internal Medicine, Infectious Diseases Service, University Medical Center Groningen, University of Groningen, P.O. Box 30.001, 9700 RB, Groningen, The Netherlands; 3 Programme de Lutte Contre la Lèpre et l′Ulcère de Buruli, Ministries of Health, Cotonou, Bénin; 4 Agogo Presbyterian Hospital, Agogo, Ghana; 5 Kwame Nkrumah University of Science and Technology, Kumasi, Ghana; 6 Department of Public Health, University Medical Center Groningen, University of Groningen, P.O. Box 196, 9700 AD, Groningen, The Netherlands; Partners for Applied Social Sciences (PASS), BELGIUM

## Abstract

Buruli ulcer (BU) is a necrotizing skin disease caused by *Mycobacterium ulcerans*. People living in remote areas in tropical Sub Saharan Africa are mostly affected. Wound care is an important component of BU management; this often needs to be extended for months after the initial antibiotic treatment. BU is reported in the literature as being painless, however clinical observations revealed that some patients experienced pain during wound care. This was the first study on pain intensity during and after wound care in BU patients and factors associated with pain. In Ghana and Benin, 52 BU patients above 5 years of age and their relatives were included between December 2012 and May 2014. Information on pain intensity during and after wound care was obtained during two consecutive weeks using the Wong-Baker Pain Scale. Median pain intensity during wound care was in the lower range (*Mdn* = 2, CV = 1), but severe pain (score > 6) was reported in nearly 30% of the patients. Nevertheless, only one patient received pain medication. Pain declined over time to low scores 2 hours after treatment. Factors associated with higher self-reported pain scores were; male gender, fear prior to treatment, pain during the night prior to treatment, and pain caused by cleaning the wound. The general idea that BU is painless is incorrect for the wound care procedure. This procedural pain deserves attention and appropriate intervention.

## Introduction

Buruli ulcer (BU) is one of the 17 Neglected Tropical Diseases, for which the World Health Organization (WHO) set their priority to improve treatment [1]. BU is highly prevalent in remote areas in Sub Saharan Africa such as Benin and Ghana and destroys skin, subcutaneous fat, and sometimes bone [2]. The disease affects all ages, but impacts predominantly on children aged 4–15 years [3,4]. Patients typically present with painless non-ulcerated lesions or ulcers with undermined edges [2,5], which are treated by antimicrobial agents (rifampicin and streptomycin), supplemented with surgical intervention if needed [6]. Even with this treatment, ulcers may last several months to over a year before they finally heal, leaving patients with scarring, calcification, contractures, and functional limitations [7,8].

Appropriate wound care is an important aspect of treatment, because it contributes to wound healing and reduces the risk of secondary infections, prolonged hospitalization and functional disabilities. Wound care is usually performed twice or thrice a week by a nurse, in a dressing room where-commonly- the patient is accompanied by a relative. This can either be in a specialized treatment center or in a local health center.

Pain during wound care, that is, *procedural pain*, is commonly observed in the clinic. Procedural pain is caused by every manipulation involving the wound [9], in contrast to background pain, which is experienced while resting. Clinical observations contrast with the literature, describing BU as a painless condition, despite the large and deep ulcers and tissue damage [10,11]. Empirical clinical studies on pain in BU are currently lacking, and there is a dire need to confirm these clinical observations. According to the WHO guidance on Buruli ulcer treatment, adequate pain relief should be provided before the dressings of large ulcers are changed however, specific information on how pain should be managed is not provided [12]. A general guidance on the basic principles of wound management in resource-poor settings could be useful to build on a Buruli ulcer-specific clinical practice guideline [13].

Self-report pain scales are the gold standard in pain assessment [14]. The Wong-Baker Faces Pain Rating Scale (WBS) is a recommended self-report measure for children ≥ 5 years of age [15]. In children aged five to seven years and those with cognitive or verbal communication deficits, however, the psychometric properties of this scale are lower [16], and an alternative scale with three faces is often used [17]. Factors affecting the patient’s pain score can be divided into stable and modifiable factors, related to the patient, disease or environment (Table 1) [18]. Generally, women report more pain than men, while previous pain experiences can work both ways; that is, by decreasing due to habituation, or increasing by sensitization. Furthermore, younger age was related to more pain behavior in a study among children aged six to 11 years [19]. The invasiveness, noxiousness and duration of the procedure are known to increase pain [18], and the anticipation of undergoing painful procedures may elevate fear or anxiety in patients, which is known to increase pain [20,21].

**Table 1:** Stable and modifiable factors related to procedural pain.

Stable factors	Patient	1) Sex
		2) Age
		3) Previous hospitalizations
	Disease	4) Duration of current hospitalization
		5) Recurrent case or not
		6) Surgery or not
		7) Type of lesion
		8) Category of lesion
		9) Location of pain
Modifiable factors	Patient	10) Fear (state)
	Environment	11) Relative’s presence
	Disease	12) Pain during removal of bandage (wet)
		13) Pain during removal of bandage (dry)
		14) Surgical debridement
		15) Type of cleaning of wound
		16) Pain during cleaning
		17) Food intake prior to treatment
		18) Pain during the night
		19) Use of pain medication

To verify the clinical observations on pain during wound care in BU, the primary objective of this study was to examine procedural pain in BU patients ≥ 5 years of age. The second objective was to examine which patient—and procedural factors influence the pain score. Besides, the psychometric properties of the WBS, and patients’ comprehension of this scale were examined.

## Methods

### Study design and ethics

This observational study used a repeated measures design. Data were collected between December 2012 and May 2014 in the Buruli ulcer treatment centers in Allada and Lalo, located in the Atlantic and Couffo regions in the south of Benin, and in the hospital in Agogo, located in the Ashanti-Akim North District in Ghana. The Beninoise National Medical Ethical Committee (ref:1961/MS/DC/SGM/DRF/ SRAO/SA) and the Ghanaian Committee of Human Research, publications and Ethics from the Kwame Nkrumah University of Science and Technology (ref:CHRPE/AP/230/12) approved the study. Data were collected once a week, for two consecutive weeks. In each of the two weeks, five assessments were performed, respectively ½ h prior to (T1), during (T2), and ½ h (T3), 1 h (T4), and 2 h (T5) after wound care. The measures in the second week were used to examine the test-retest reliability of the pain scales.

### Setting

Data were collected in three specialized BU treatment centers; Lalo, Allada, and Agogo. These three centers are specialized for BU treatment, and the main referral center for local health posts. The referral centers provide diagnostic procedures, antibiotic treatment, and surgical services if needed, in accordance with WHO guidelines [12]. Although variation exists between different settings [22], generally, wound care is performed twice or thrice per week by a nurse, in a dressing room where the patient is accompanied by a relative. The bandage is removed by the patient or the relative, the wound is cleaned by the attending nurse with water or normal saline, or solutions like hypochlorite, Betadine or metronidazole, after which new bandages are applied. This repetitive procedure takes place throughout the entire admission period, until the wound has healed. The dressing room is cleaned daily and well equipped with dressing material, cleaning products and gloves.

## Participants

One day before the intended interview, all BU patients who were clinically diagnosed in line with the definitions of the World Health Organization [23], and above five years of age, were approached together with their relatives and informed about the study. Written informed consent was documented from all patients > 18 years of age, and parents of patients < 18 years of age. In addition, written assent was obtained from patients between 10 and 18 years of age, in accordance with principles of Good Clinical Practice. Relatives of patients aged 5–7 years were included as proxy report for the patients, since the ability of the young patients to use pain scales was unclear beforehand. Thus, the relatives provided additional information about the young patient’s pain. Patients not attending wound care or scheduled for surgery on the study day were excluded.

### Data collection and procedure

Data were collected through interviews using questionnaires, conducted in the hospital, in a private setting at the ward, or in the waiting area outside the dressing room. Interviews were conducted with patients and relatives separately. During the interview at T1 (½ h prior to treatment), socio demographic information was collected (sex, age, ethnicity, level of education), as well as information on previous hospital admissions, previous pain experiences (assessed by closed (yes/no) questions) and pain during the past night. Pain was assessed at all five assessment points (T1, T2, T3, T4, T5). Fear was assessed at T1. Wound characteristics and procedural factors were assessed at T2 by a checklist for each patient, filled in by a research assistant in collaboration with a treating nurse. A pain scale accuracy task was proposed at T5 to examine the ability of patients to use the pain scales.

#### Interviewers and translation of questionnaires

Native interviewers, experienced with questionnaire-based research and able to speak the local languages in the study sites in Benin and Ghana, collected the data. At the start of the study, they engaged in a training program covering the study procedures, explanation of the questionnaires, and potential biases.

The interviews in Benin were conducted in Fon or Adja. The official language in Benin is French, thus, all questions were first translated from English to French by a translator fluent in both languages, and then orally into Fon by the interviewer. An exception to this was the WBS, for which the official French translation was used and translated into Fon. In Ghana, interviews were conducted in Twi or other local language in the study site in Ghana. The official language in Ghana is English, thus, all questions were orally translated from English into Twi. The official English translation of the WBS and the Children’s Fear Scale were used and translated into Twi.

### Measures

#### Self—reported pain: Wong Baker scale

The WBS was administered to the patients to assess self-reported pain intensity, and to the relatives of patients < 7 years of age to examine the validity of the responses of the young patients to the 3-faces scale (described in the next paragraph). The WBS is a one item horizontal scale of 6 hand-drawn faces, scored from 0 to 10, ranging from a smiling “no hurt” face on the left to a crying “hurts worst” face on the right. The WBS has undergone extensive psychometric testing of the validity (construct, known group) and reliability (test-retest, concordance with observational score, responsiveness) and was used in both acute and disease related pain in children between 3–18 years of age without cognitive impairment [15].

#### Self—reported pain: 3-faces scale

The 3-faces scale was used to assess self-reported pain intensity in patients between five and seven years of age. This response scale was developed for the PedsQL 4.0 Generic Core scale (a pediatric quality of life questionnaire), as an alternative to the 5-point Likert scale for young children (ages 5–7) with different conditions [17]. It is a simple 3-point scale (1 = no pain, 2 = a bit of pain, 3 = a lot of pain), with each response choice anchored from a happy to a sad face.

#### Stable and modifiable factors related to pain

Stable factors (1 to 9) and modifiable factors (10, 11, 18 and 19) (see Table 1), were assessed by a questionnaire ½ hour prior to treatment (T1). These included patient factors, such as sex, age, fear (explained below), and previous hospitalizations, as well as disease factors such as food intake prior to treatment, pain during the night and the use of pain medication.

Stable factors 7 and 8, and modifiable factors 12 to 17 (Table 1), were assessed by a checklist during treatment. Wound characteristics, that is the type (plaque, nodule, edema, ulcer < 10 cm, ulcer > 10 cm, critical site, osteomyelitis) and category (1, 2 or 3 [24]) of the lesion were noted. Removal of the bandage (wet/dry), cleaning of the wound (yes/no), type of cleaning material, surgical debridement and covering of the wound, as well as the rated pain-intensity scores (0–10) associated with each of these factor was noted.

#### Fear

Fear was assessed using the Children’s Fear Scale (CFS); a one-item self-report measure consisting of five hand-drawn sex-neutral faces. This scale was validated in children between 5 and 10 years of age, supporting construct validity, inter-rater reliability and test-retest reliability of the measure [25]. This scale was used for all patients, including patients above 10 years of age, because there are limited validated self-report tools to measure fear in busy, clinical settings.

### Data analysis

Data were analyzed using SPSS version 20.

#### Psychometric analyses of the WBS

The psychometric quality of the WBS was examined in our study population by investigating the acceptability, concurrent validity, responsiveness and test-retest reliability of the scale. Acceptability of the WBS was evaluated by 11 health professionals involved in BU treatment as part of a parallel study for which the manuscript is currently in preparation. Concurrent validity was tested by comparing the WBS with the widely used Faces Pain Scale Revised (FPS-R), which is a one item self-reported pain measure, comprising 6 sex-neutral faces of increasing levels of pain. The FPS-R has shown to have good psychometric properties in pediatric patients [15,26,27]. The concurrent validity of the WBS was tested by examining the agreement between patients’ responses on the WBS and the FPS-R by a two way, fixed effect model Intra Class Correlation single measures/absolute agreement (ICC_1,A_). Responsiveness, that is the ability of the WBS to detect differences between a painful (T2: during treatment) and a relatively non-painful (T5: 2 hours after treatment) situation, was examined using a Wilcoxon Signed-Rank test. A mean difference of two points on the WBS between T2 and T5 was considered to be meaningful [15]. Test-retest reliability of the WBS was examined by comparing pain scores of patients between week 1 and week 2 at T1, T2, T3, T4 and T5.

#### Psychometric analyses of the 3-faces scale

Pain scores of the patients between five and seven years of age on the 3-faces scale were transformed to a 0–10 scale (0 = no pain, 5 = a bit of pain, 10 = a lot of pain)—a transformation which has been used previously [17]—in order to compare the 3-faces scores of the young patients to the WBS scores of their relatives. Since a gold standard is lacking in this situation, convergent construct validity was used to provide evidence for the validity of the 3-faces scale scores of the young patients [28]. A Pearson’s correlation of 0.64 between the pain report of parent and child was found previously in a large meta-analysis, and was considered to be satisfactory construct validity [29]. Since Pearson’s r does not take into account systematic errors, a more critical parameter, namely the ICC_1, A_, was used [30]. At each time point (T2 to T5), the ICC_1, A_ was 0.92, 0.72, 0.62 and 0.54 respectively, supporting the construct validity of the 3-faces scale scores for the young patients. Therefore, the transformed 3-faces scores of the young patients were used to describe the pain intensity in the young age group.

#### Accuracy check for the pain scales

An accuracy task was proposed to check whether patients and relatives were able to accurately use the WBS, or when appropriate, the 3-faces scale. This gave insight into whether participants understood the pain scales. Accuracy was determined by considering whether the judgments of pain severity of respondents matched the pain severity depicted in three vignettes, a method which was derived from Stanford et al. (2006) [31]. The first vignette addressed no pain (playing with friends at home); the second vignette indicated low pain (being pushed down by a play mate); the third vignette showed severe pain (burning hand on stove at cooking place).

All patients, and relatives of patients between 5 and 7 years of age, were presented with the three vignettes. Patients above 7 years of age and the relatives of patients between 5 and 7 years of age responded to the WBS, while the patients between five and seven years of age responded to the 3-faces scale. The expected score on the WBS for the vignette expressing no pain was 0 or 2, for the vignette expressing mild pain 2, 4 or 6, and for the vignette expressing severe pain 6, 8 or 10. The expected score on the 3-faces scale was 1 for the first vignette, 2 for the second vignette, and 3 for the third vignette.

Descriptive statistics were used to assess patient and relative characteristics. The data on pain (WBS, 3-faces scale) and fear (CFS) were ordinal (6-point intensity scales) and non-normally distributed; scores showed a positively skewed distribution. Therefore, the median (*Mdn*), interquartile range (*IQR*) and Coefficients of Variance were used to describe pain intensity reported by the WBS. The coefficient of variation (*COV*) for ordinal variables was used as a standardized measure of precision, based upon the IQR (25^th^ percentile- 75^th^ percentile/*Mdn*) [32]. Mann-Whitney U tests and Kruskall-Wallis tests statistics were used to examine the relationship between pain intensity scores and categorical explanatory variables, and Spearman’s rank correlation was used to examine correlations between pain intensity scores and continuous explanatory variables. Due to unequally spaced time intervals, a mixed model analysis was used to examine the change in pain scores over time. Time was included as a factor with 5 time points. The residuals were checked for their normal distribution.

## Results

### Patients characteristics

The response rate was 98%: 52/53 agreed to participate. Fifty-two patients were interviewed in week one, of which 49 patients were followed up in week two. Patients dropped out because of healing of the wound (n = 2) or because treatment was performed in another center (n = 1). Twelve patients (23%) were between five and seven years of age, and responded to the 3-faces scale instead of the WBS. Their attendant was mostly the mother (n = 8), the father (n = 2) a sister or an acquaintance. Irrespective of the type of relationship to the patient, the attendant took care of the patient by cooking, washing laundry, cleaning and more. 24 patients were female (46%), and the mean age was 16.3 years (SD = 14.3). Two patients reported to have taken pain medication prior to wound care, which was either paracetamol or diclofenac.

### Validity and reliability of the Wong Baker scale

Of the 11 interviewed professionals involved in BU treatment [manuscript in preparation], 5 preferred the Wong-Baker scale, 3 preferred the Faces Pain Scale-Revised, and 1 found both equally useful, supporting acceptability of the Wong-Baker scale. This is consistent with previous studies [15]. The agreement between the WBS and the FPS-R at the time points T1, T2, T3, T4 and T5 ranged between 0.97 and 1.00, well above the acceptable threshold value of 0.70 [33], supporting concurrent validity. Responsiveness was satisfactory, as indicated by a significant difference in medians between T2 and T5 of 2 points. The agreement between the pain scores in week 1 and week 2 (ICC_1, A_) for each time point (T1 to T5) were 0.70, 0.72, 0.70, 0.81, and 0.49 respectively, thus, test-retest reliability was satisfactory, except for the scores on T5 (2h post-treatment). We controlled for change in procedural factors between week 1 and week 2, and found no differences in soreness of removing the bandage dry (ICC_1, A_ = 0.86) removing the bandage with water (ICC_1, A_ = 0.95) and soreness of covering the wound (ICC_1, A_ = 0.93). Clinical characteristics of the patient were recorded, and there was only one patients who had surgery between week 1 and week 2, who was therefore excluded for the retest measurement.

### Pain scale accuracy task

The first vignette was rated as ‘no pain’ (*Mdn* = 0; *CV* = 0), the second vignette as ‘mild pain’ (*Mdn* = 4; *CV* = 0.5) and the third vignette as ‘severe pain’ (*Mdn* = 6; *CV* = 0.33). Thus, patients’ and relatives’ comprehension of the WBS was sufficient, although the vignette expressing severe pain was scored on the low side. For the 3-faces scale, the first vignette was rated as ‘no pain’ (*Mdn* = 1; *CV* = 1), the second vignette as ‘mild pain’ (*Mdn* = 2; *CV* = 0) and the third vignette as severe pain (*Mdn* = 3; *CV* = 0.33). We conclude that scale comprehension of the 3-faces scale was sufficient in the age group five to seven years.

### Pain intensity scores prior to, during and after treatment

**Fig 1:**
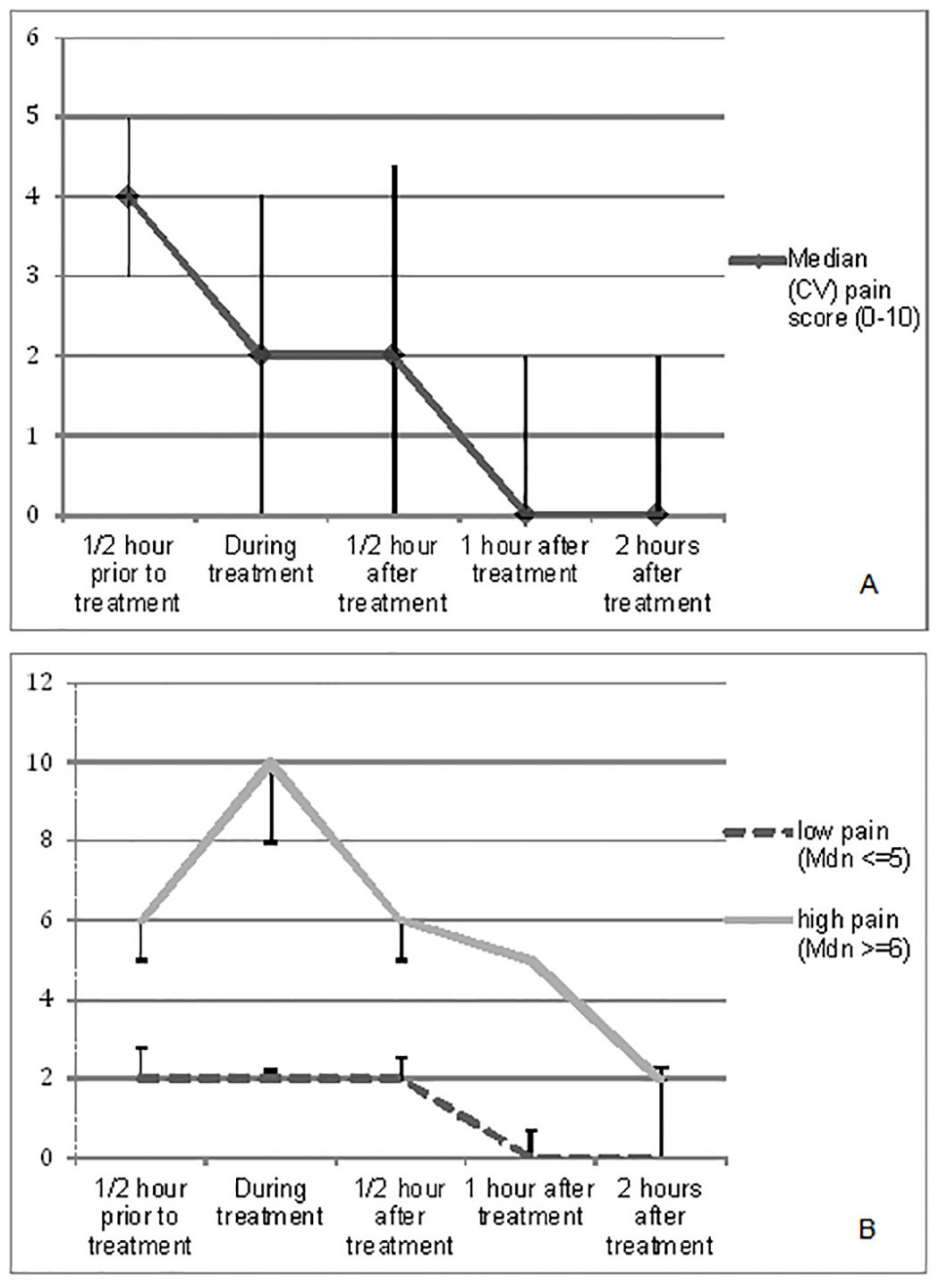
Median (CV) pain intensity scores (range 0–10) over time (A), and pain scores reported by patients with high (n = 14) and low (n = 38) pain during treatment (B).

Pain intensity scores of patients are presented in Fig 1(A), with scores in the midrange half an hour prior to (*Mdn* = 4, CV = 1), and in the lower range during treatment (*Mdn* = 2, CV = 1). Pain scores declined significantly over time towards nearly no pain two hours after treatment (*F* (4, 21) = 16.28, *p* <.001). Fig 1(B) shows that 27% (*n* = 14) of patients reported high pain scores (> 6) during treatment, and scores declined over time. The decline in pain scores over time was statistically significant (*F* 4 = 36.40, *p* <.001).

### Patient- and wound characteristics and procedural factors, and relationships with pain

The relationships between patient characteristics and pain during treatment are presented in Table 1, and between stable and modifiable variables and pain during treatment in Table 2 and Table 3, respectively. Higher pain scores were found in males (*Mdn* = 4, *CV* = 2) compared to females (*Mdn* = 2, *CV* = 1.75, *U* = 187.0; *p* = .025). Furthermore, higher fear prior to treatment (*r*_*s*_ = .50, *p* <.001), pain during the night prior to wound care (*r*_*s*_ = .64, *p* <.001) and removing the bandage dry (*r*_*s*_ = .74, *p* <.004) were related to higher pain scores during treatment. Pain during cleaning of the wound was related to higher pain scores (*r*_s_ .68, *p* = <.001), however, the type of cleaning; water or normal saline, or other types, such as hypochlorite, Betadine or metronidazole, was not related to pain during treatment (*U* = 146.5, *p* = .77).

**Table 2:** Stable factors, and relationships with pain during treatment (measured by the Wong Baker scale).

Factor	Value (s)	N (% of total)/ Mdn (CV)	R_s_ *	*p*	N
1) Sex	Male, pain intensity (0–10)	4 (2)		.005**	52
	Female, pain intensity (0–10)	2 (1.75)			
2) Age		16.3 (14.3)	-.10	.47	52
3) Pain previous hospitalizations	Yes, n (%)	14 (42.4)		.03**	33
4) Duration of current hospitalization	Weeks, Mdn (CV)	16 (14.1)	-.12	.40	49
5) Recurrent case	Yes, n (%)	22 (42,3)		.36**	52
6) Surgery	Yes, n (%)	18 (34,6)		.67**	52
7) Type of lesion	Plaque	1 (2.5)		.17***	40
	Ulcer < 10 cm	13 (32.5)			
	Ulcer > 10 cm	25 (62.5)			
	Osteomyelitis	1 (2.5)			
8) Category of lesion	1	9 (18.4)		.06***	49
	2	10 (20.4)			
	3	30 (61.2)			
9) Location of pain	Upper limb	7 (13.5)		.55***	23
	Lower limb	14 (26.9)			
	Abdomen	1 (1.9)			
	Back	1 (1.9)			

* Rs = Spearman's rank correlation,

** Mann-Whitney U test

*** Kruskall-Wallis test

**Table 3:** Modifiable factors, and relationship to pain during treatment (measured by the Wong Baker scale).

Factor	Value (s)	N (%)/ Mdn (CV)	Rs*	P	N
10) Fear intensity prior to treatment	Intensity (1–5)	2 (1)	.50	<.001	52
11) Presence relative	Yes, n (%)	41 (79)		.30**	52
12) Removal of bandage, only wet	Intensity (0–10)	4 (2.5)	.29	.45	9
13) Removal of bandage, only dry	Intensity (0–10)	1 (3.5)	.74	.004	13
14) Surgical debridement	Yes, n (%)	4 (7.7)		.83**	52
15) Cleaning of wound	Water/ normal saline	45 (86.5)		.77**	52
	Other (hypochlorite, Betadine, metronidazole)	7 (13.5)			
16) Cleaning	Intensity (0–10)	1 (4.5)	.68	<.001	33
17) Food intake before procedure	Yes, n (%)	22 (42,3)		.84**	52
18) Pain during the night	Intensity (0–10)	4 (1)	.64	<.001	35

* Rs = Spearman's rank correlation

** Mann-Whitney U test

## Discussion

This is the first study describing pain associated with wound care treatment, and factors related to pain in Buruli ulcer. By using self-reported pain scales, this study showed that pain prior to and during wound care was in the midrange, with severe pain (scores > 6 during treatment) reported in nearly 30% of the patients. Pain declined over time towards nearly no pain reported one and two hours after completion of wound care. Factors associated with higher pain scores during treatment were; being male, fear prior to treatment, pain during the night prior to the wound care, and pain while cleaning the wound.

Our findings are a useful addition to the literature on pain in BU, which has predominantly stressed the typical lack of pain of the condition at presentation. This lack of pain is suggested to occur as a result of the pathogen *Mycobacterium ulcerans* producing *mycolactone* [34,35]. Mycolactone has been suggested to cause the nerve damage that is responsible for the painlessness in BU [11,36]. Antimicrobial treatment leads to a decrease in mycolactone production 6 to 12 weeks after the start of treatment [37]. Our findings suggest that pain in association with wound care is common in Buruli ulcer. Nerve regeneration during the healing process of the wound might explain why patients experienced pain over the course of their treatment. A report by Nienhuis et al. also suggested a recovered sensation, since the BU patients sometimes complained of pain at the lesion site just before and at the time the lesion ulcerated [38]. However, the current study did not address the process of nerve regeneration, nor a drop of mycolactone concentration over time.

This study showed that pain intensity half an hour prior to wound care was significantly higher than pain during wound care. Our interpretation is that both the pain score half an hour *prior to* and the pain score *during* wound care were in part confounded by anticipatory fear. With respect to the pain score during wound care, this interpretation is supported by the significant correlation between fear prior to treatment and pain during treatment. On the other hand, the gradual decrease in pain over time argues against this confounding effect. If the pain score during treatment would actually be fear, the drop in pain after treatment would probably be steeper. For the pain score prior to treatment, it is more likely that fear and pain were contaminated, which is in concordance with literature on the moderate to strong relationship between fear and pain [25]. This study did not separate the effect of fear from the effect of pain on the explanatory variables, which would be an interesting avenue for future research.

Besides fear prior to treatment, higher pain scores were found in males compared to females, a contrasting finding to the literature, since females typically report higher pain scores compared to males [18]. Also in this study area, it is suggested that males are expected to report less pain, compared to women [manuscript in preparation]. This contrasting finding could be a result of the relatively small sample size. Third, pain during cleaning the wound, and removing the bandage dry was related to higher self-reported pain scores. This is in line with clinical observations, [22], suggesting that this is a painful aspect of treatment. The type of cleaning product, did, however, not affect the pain score. It is important to note that there was no effect of age: pain was prevalent across all age ranges of BU patients, which is contrasting the literature that children generally report higher pain scores compared to adults [18]. Fourth, pain during the night was related to higher pain scores during treatment, indicating that there seemed to be background pain in BU patients. It should be noted that this question was proposed to the patient half an hour prior to treatment, which might have contributed to a biased response about the pain experience during the night.

This study showed high pain in a subgroup of patients, and several modifiable factors to be related to higher pain scores, such as fear prior to treatment and pain while cleaning the wound. A next step would be to examine the development of pain throughout the disease process in a larger, longitudinal study in which patients are followed throughout the period of hospitalization. Furthermore, relating the results of pain to the M. ulcerans and mycolactone production could provide insights into the underlying mechanism. That being said, results provide directions for a change of future daily practice.

First of all, despite the finding that a subgroup of patients reported high (≥ 6) pain scores, none of the patients received pain medication prior to treatment. The indication, type and dose of pain medication should be discussed. Secondly, pain while cleaning the wound was related to higher pain during treatment. This could be related to the fact that in current practice, health professionals sometimes remove necrotic tissue while cleaning the wound without sedation and / or pain killers. This finding should be discussed in medical teams treating BU. Thirdly, the finding that fear was related to more pain, implies that a non-pharmaceutical intervention to reduce anticipatory fear could be explored. There is a large array of non-pharmaceutical interventions available from the treatment of burn wound pain. An option could be to provide procedural and sensory information at the first day of arrival at the treatment center. Furthermore, providing a booklet, educating the family, learning positive self-talk, using filmed modeling, and distraction during treatment are possible interventions.

To arrive at a change in future daily practice, a standardized pain protocol for BU could be developed. A parallel study on health personnel’s attitudes and beliefs about pain assessment and treatment, and current practices in prescribing pain medication in BU provides additional input to the current study for such a protocol [manuscript in preparation]. A collaboration between the National programs fighting BU in endemic countries, health professionals treating BU from different treatment centers, and experts on pain treatment is therefore essential.

No differences in pain scores were found between patients who are in different phases of their wound care treatment. This phase was estimated by the duration of admission, lesion type and whether patients had a surgical intervention. Also, the stage of healing of the wounds was estimated by looking at three aspects, namely, the duration of hospitalization, the type of lesion, and whether patients had undergone surgery recently. None of these factors influenced the pain intensity reported by the patients.

Mycolactone is responsible for the painlessness in BU. Recently, the mechanism was suggested to be a hyperpolarization of neurons instead of direct nerve damage [10,11]. During antimicrobial treatment, the mycolactone production is lowered and therefore, analgesia is expected to reduce, and consequently, to increase pain during treatment. Other factors in the wound care e.g. epithelialization or fear may have masked this effect.

Strengths of this study were the satisfactory reliability (test-retest) and validity (face validity, construct validity, responsiveness) of the WBS in this new patient population, contributing to the quality of our findings. Furthermore, the construct validity of the 3-faces scale was demonstrated by the high agreement among scores of the young patients and their relatives at the same time point. Third, a pain scale accuracy task, testing the patient’s scale comprehension, demonstrated the ability of the patients to use the pain scales. Finally, the study was carried out in a real-life setting, and did not interrupt the usual care, leading to a realistic picture of current practice of BU wound care in two different, endemic settings in rural Africa.

A limitation of the study is the heterogeneous sample of BU patients included, with a wide range in age, clinical manifestation (plaque, edema, ulcer) and stage of healing. This, in combination with the relatively small sample size for each subset of patient groups, might have led to statistically insignificant associations between explanatory variables and the outcome. Second, as said before, the lack of a good indicator for the stage of wound healing, made it difficult to examine the relationship between this factor and pain, as well as exploring pain throughout the treatment process. Third, by introducing pain scales, a response bias might have been introduced. The current clinical practice has not included pain assessments, thus, talking about-, and asking about pain might have introduced bias, such as changing expectations about treatment.

In conclusion, our study was the first to address pain in conjunction with wound care in BU patients. Reported pain intensity was commonly in the mid-range; and severe pain was reported in nearly 30% of BU patients. This procedural pain deserves attention and appropriate intervention, which could be examined in future studies on possibilities to reduce pain during treatment for BU.
